# The status of and future research into Myalgic Encephalomyelitis and Chronic Fatigue Syndrome: the need of accurate diagnosis, objective assessment, and acknowledging biological and clinical subgroups

**DOI:** 10.3389/fphys.2014.00109

**Published:** 2014-03-27

**Authors:** Frank N. M. Twisk

**Affiliations:** ME-de-Patiënten FoundationLimmen, Netherlands

**Keywords:** Myalgic Encephalomyelitis, Chronic Fatigue Syndrome, assessment, diagnosis, immune system, post-exertional malaise, subgroups

## Abstract

Although Myalgic Encephalomyelitis (ME) and Chronic Fatigue Syndrome (CFS) are used interchangeably, the diagnostic criteria define two distinct clinical entities. Cognitive impairment, (muscle) weakness, circulatory disturbances, marked variability of symptoms, and, above all, post-exertional malaise: a long-lasting increase of symptoms after a minor exertion, are distinctive symptoms of ME. This latter phenomenon separates ME, a neuro-immune illness, from chronic fatigue (syndrome), other disorders and deconditioning. The introduction of the label, but more importantly the diagnostic criteria for CFS have generated much confusion, mostly because chronic fatigue is a subjective and ambiguous notion. CFS was redefined in 1994 into unexplained (persistent or relapsing) chronic fatigue, accompanied by at least four out of eight symptoms, e.g., headaches and unrefreshing sleep. Most of the research into ME and/or CFS in the last decades was based upon the multivalent CFS criteria, which define a heterogeneous patient group. Due to the fact that fatigue and other symptoms are non-discriminative, subjective experiences, research has been hampered. Various authors have questioned the physiological nature of the symptoms and qualified ME/CFS as somatization. However, various typical symptoms can be assessed objectively using standardized methods. Despite subjective and unclear criteria and measures, research has observed specific abnormalities in ME/CFS repetitively, e.g., immunological abnormalities, oxidative and nitrosative stress, neurological anomalies, circulatory deficits and mitochondrial dysfunction. However, to improve future research standards and patient care, it is crucial that patients with post-exertional malaise (ME) and patients without this odd phenomenon are acknowledged as separate clinical entities that the diagnosis of ME and CFS in research and clinical practice is based upon accurate criteria and an objective assessment of characteristic symptoms, as much as possible that well-defined clinical and biological subgroups of ME and CFS patients are investigated in more detail, and that patients are monitored before, during and after interventions with objective measures and biomarkers.

## Introduction

Myalgic Encephalomyelitis (ME) and Chronic Fatigue Syndrome (CFS) have been marred by much controversy (Holgate et al., 2011), from the authenticity of the symptoms (Chalder et al., 1996; Carruthers et al., 2011) and the existence of the ME and CFS as distinct clinical entities (Barsky and Borus, 1999; Twisk and Arnoldus, 2013) to the etiology (Huibers and Wessely, 2006; Carruthers et al., 2011), the pathophysiology (Vercoulen et al., 1994; Maes and Twisk, 2010) and presumed effective interventions, e.g., cognitive behavioral therapy (CBT) and graded exercise therapies (GET) (Knoop et al., 2007; Twisk and Arnoldus, 2012).

An important part of the controversy originates from the introduction of the label CFS, but even more from the definitional criteria of this clinical entity (Fukuda et al., 1994), which define a heterogeneous population of people of chronic fatigue (Wilson et al., 2001), and the use of self-reported subjective measures for ambiguous and insignificant symptoms like “fatigue” (Jason et al., 2009). The principle feature of CFS is (unexplained) chronic fatigue (Fukuda et al., 1994).

ME, whether defined by the original criteria (Ramsay, 1988) or the proposed new criteria (Carruthers et al., 2011), is not equivalent to CFS (Fukuda et al., 1994), let alone a severe form of incapacitating chronic fatigue (Sharpe et al., 1991; Reeves et al., 2005). Distinctive symptoms of ME are profound (muscle) weakness and cognitive deficits (“brain fog”). More importantly, ME can be distinguished from other diseases, chronic fatigue and deconditioning by post-exertional “malaise”: a prolonged intensification of symptoms, e.g., “brain fog” and muscle pain after a minor exertion. This distinctive phenomenon is reflected by a dramatic fall in maximum oxygen uptake and oxygen uptake and workload at the aerobic threshold at a second exercise test 24 h after the first (Snell et al., 2013) and a long-lasting increase of muscle metabolite-detecting receptors for pain and “fatigue” after moderate exercise (White et al., 2012).

Methodological issues, e.g., symptom-based criteria, the subjectivity of the symptoms, mixing patients with post-exertional “malaise” and abnormalities (ME) and patients without this phenomenon (CFS/not ME) in research and clinical practice, and assessment of the clinical status and improvement thereof in trials of proposed effective therapies using subjective measures, have yielded contradictory results and have fuelled the debate.

Despite the heterogeneity of the patient population, due to the equivocal CFS criteria, various abnormalities have been observed repetitively in the CFS patient group as a whole or in substantial subgroups of the CFS population. However, scientific progress has been slow and unnecessarily obstructed by the methodological obstacles mentioned above. In addition, due to the heterogeneity of the patients studied selected by CFS criteria, it is highly unlikely that future research will reveal one or more abnormalities that are applicable to all CFS patients.

To surmount the impasse, which has lasted for decades, it is essential to improve the standards of the research into ME/CFS, it is crucial to leave the current methods behind us and to assess symptoms objectively as much as possible, to make a clear distinction between patients with and without post-exertional abnormalities, e.g., by employing repeated cardiopulmonary exercise tests, to accurately diagnose patients using ME and CFS criteria, to define clinical and biological ME and CFS patient subgroups in research studies, and to monitor patients before, during and after trials using objective measures, however, trivial this may seem. This partly due to the fact that, in contrast with other illnesses, ME and CFS are not yet clearly defined by distinctive biomarkers. Without doing so, the debate around ME/CFS will persist.

This article summarizes the current status and is directed to the abnormalities established repeatedly and the potential improvement of diagnosis of and research into ME and CFS.

## Criteria

### ME (1934)

Outbreaks of a disease resembling “atypical poliomyelitis” (Gilliam, 1938) occurred from the 1940s through the 1980s at various places over the world (The Medical Staff of The Royal Free Hospital, 1957; 1978; Hyde et al., 1992). ME was identified as a new clinical entity in 1959 (Acheson, 1959) and has been acknowledged as a disease of the central nervous system/neurological disease by the World Health Organization since 1969 (WHO, 1967). The clinical picture was defined by Ramsay and co-workers (Ramsay, 1988; Dowsett et al., 1990; Jason et al., 2012a) in the 80 s. Distinctive symptoms of ME (Goodwin, 1981) are: profound (muscle) weakness and tenderness, easy fatigability of the muscles, neurological abnormalities, e.g., visual problems, cognitive deficits (concentration and memory problems), circulatory disturbances, and post-exertional “malaise”: a (delayed) long-lasting increase of symptoms after a minor physical and mental exertion.

### CFS (1994)

The diagnostic entity CFS was introduced in 1988 (Holmes et al., 1988) after an outbreak in Nevada at Lake Tahoe in the mid-eighties. CFS was redefined in 1994 (Fukuda et al., 1994) into clinically evaluated, unexplained (persistent or relapsing) chronic fatigue, accompanied by at least four out of a list of eight symptoms, e.g., sore throat, unrefreshing sleep, and headaches. The majority of research into ME/CFS^1^ in the last two decades is based upon these case criteria. Since “fatigue” and other symptoms, e.g., unrefreshing sleep, are subjective, non-specific and ambiguous, the CFS criteria select a varied population of people with chronic fatigue (Wilson et al., 2001).

### ME (2011)

The heterogeneity of the CFS (Fukuda et al., 1994) patient population has impeded effective research and accurate diagnosis of patients. For that reason a group of researchers have proposed criteria (Carruthers et al., 2011) in order to distinguish ME from CFS (Fukuda et al., 1994) and chronic fatigue (Sharpe et al., 1991). According to this new criteria post-exertional “malaise” or “neuro-immune exhaustion” (“a pathological inability to produce sufficient energy on demand” resulting into symptom exacerbation, e.g., flu-like symptoms and pain, after minor exertion) is obligatory for the diagnosis ME. Post-exertional “malaise” should be accompanied by specific neurological symptoms, impaired cellular energy metabolism and/or transportation, and immune, gastro-intestinal or genitourinary symptoms.

### ME vs. CFS

Although ME and CFS are considered to be interchangeable labels (WHO, 1992), criteria for ME (Carruthers et al., 2011) and CFS (Fukuda et al., 1994) define distinct, partially overlapping clinical entities (Twisk and Arnoldus, 2013). Post-exertional malaise and cognitive deficits e.g., are not mandatory for the diagnosis CFS, while these symptoms are obligatory for the diagnosis ME. “Fatigue” is not obligatory to meet the diagnosis ME. The distinction between patients with ME/CFS with post-exertional malaise and patients without post-exertional malaise is reflected by particular clinical and immunological differences (Maes et al., 2012a; Brenu et al., 2013).

### Prevalence and impact

Nacul et al. (2011) found that 0.19% of 143,000 individuals (18–64 years) met the commonly used Fukuda criteria for CFS (Fukuda et al., 1994), while 0.11% met the more strict criteria for ME/CFS (Carruthers et al., 2003), including post-exertional malaise. Prevalence rates of ME (Carruthers et al., 2011) remain to be investigated, but based upon (Nacul et al., 2011; Jason et al., 2012b; Maes et al., 2012a; Brenu et al., 2013) it is estimated that 30–50% of the subjects meeting the CFS (Fukuda et al., 1994) criteria fulfill the more stringent criteria for ME (Carruthers et al., 2011). Patients experience significantly more impairment than patients with hypertension, congestive heart failure, type II diabetes mellitus, acute myocardial infarction, multiple sclerosis and depression (Komaroff et al., 1996). The ME/CFS criteria (Carruthers et al., 2003) select patients with more disability and more physical, mental and cognitive symptoms than patients with the diagnosis CFS (Fukuda et al., 1994) not meeting these criteria (Jason et al., 2012b).

## Diagnosis

Nowadays, the diagnoses ME and CFS are often based upon subjective measures reported by patients, e.g., fatigue (Chalder et al., 1993; Vercoulen et al., 1994) and physical functioning (Ware and Sherbourne, 1992; McHorney et al., 1993). However, to bypass problems created by ambiguous symptom-based criteria, e.g., heterogeneity of the patient population, a clinical assessment ME of CFS and its severity should be based upon objective measures as much as possible. Several distinctive symptoms can be assessed objectively (see Table 1).

**Table 1:** **Symptoms and tests**.

**Symptom**	**Test**	**References**
Loss of energy/weakness	Cardiopulmonary exercise test (CPET) (American College of Sports Medicine, 2009; Balady et al., 2010)	De Becker et al., 2000; Farquhar et al., 2002; Jones et al., 2012
Cognitive deficits	Specific neurocognitive tests^*^ (Wechsler, 1981; Cambridge Cognition, 1999; Lezak et al., 2004; Strauss et al., 2006)	DeLuca et al., 1993, 2004; Tiersky et al., 1997; Dickson et al., 2009; Thomas and Smith, 2009; Cockshell and Mathias, 2010; Constant et al., 2011
Muscle weakness	Muscle (power and endurance) tests (Van der Ploeg, 1991; Andrews et al., 1996; Wang et al., 2002; Stark et al., 2011)	Paul et al., 1999; Fulcher and White, 2000; Lawrie et al., 2000; Siemionow et al., 2004
Orthostatic intolerance	Tilt table test (Streeten, 1987; American College of Cardiology et al., 1996; Task Force for the Diagnosis and Management of Syncope, 2009)	Rowe et al., 1995; De Lorenzo et al., 1997; Streeten and Anderson, 1998; Stewart et al., 1999; Newton et al., 2007; Galland et al., 2008; Hoad et al., 2008; Katz et al., 2011
Post-exertional malaise		
Physical	Repeated cardiopulmonary exercise tests, 24 h apart (Katch et al., 1982; Amann et al., 2004; Bensimhon et al., 2008; Balady et al., 2010)	VanNess et al., 2006; Patrick Neary et al., 2008; Vermeulen et al., 2010; Snell et al., 2013
Cognitive	Specific neurocognitive tests^*^ (Wechsler, 1981; Cambridge Cognition, 1999; Lezak et al., 2004; Strauss et al., 2006), before and after a CPET or orthostatic stress	VanNess et al., 2007; Ocon et al., 2012
Visual symptoms	Useful field of view tests (Ball et al., 1993; Ball and Owsley, 1993) and eye movement tests (Rommelse et al., 2008)	Leslie, 1997; Vedelago, 1997; Badham and Hutchinson, 2013; Hutchinson and Badham, 2013
Sleep disturbances	Polysomnografic investigation (Rechtschaffen and Kales, 1968; Dumermuth et al., 1983; Lo et al., 2002; Iber et al., 2007)	Kishi et al., 2008, 2011; Decker et al., 2009
Defective stress response	Hormonal investigation (Kirschbaum et al., 1993; Holtorf, 2008; Kovacs and Ojeda, 2011; Melmed et al., 2011)	MacHale et al., 1998; Gaab et al., 2002; Cleare, 2004; Jerjes et al., 2005; Holtorf, 2008; Torres-Harding et al., 2008; Jammes et al., 2009; Papadopoulos and Cleare, 2011; Tak et al., 2011

* *Cognitive impairments can be identified if appropriate measures/tests are used (Thomas and Smith, 2009; Cockshell and Mathias, 2010)*.

Symptoms that cannot be assessed objectively easily due to their nature, are: “fatigue”, widespread muscle and/or joint pain, often amplified after a (minor) exertion, hypersensitivity to light, sound, odor and chemicals, e.g., anaesthetics (likely due to “central sensitization”), thermoregulation problems and “sickness behavior.” Research (Jason et al., 2014) has shown that minimum frequency and severity thresholds should be used for self-reported symptoms to reduce the likelihood of misclassification.

## Abnormalities

The heterogeneity of the patient population (Whistler et al., 2003) and the use of various methods and samples have contributed to conflicting results. Despite this various specific abnormalities have been observed repetitively in the ME/CFS patient population or substantial subgroup thereof (Table 2).

**Table 2:** **Abnormalities in ME/CFS**.

**Abnormality**	**References**
Immunological aberrations (inflammation, immune activation, immunosuppression and immune dysfunction);	Klimas et al., 1990; Fletcher et al., 2009; Lorusso et al., 2009; Meeus et al., 2009; Brenu et al., 2011; Maes et al., 2012b
consistent with processes observed during (latent) infection;	Lloyd et al., 1993; Kerr et al., 2008a; Broderick et al., 2010
Intestinal dysbiosis, inflammation and hyperpermeability,	Maes et al., 2007a; Sheedy et al., 2009; Lakhan and Kirchgessner, 2010; De Meirleir et al., 2013; Frémont et al., 2013
associated with systemic immune system abnormalities;	Maes et al., 2012c; Groeger et al., 2013
(reactivating and/or persistent) infections;	Hilgers and Frank, 1996; Chia and Chia, 2003; Nicolson et al., 2003; Chia et al., 2010; Chapenko et al., 2012
Elevated oxidative and nitrosative stress;	Zhang et al., 1995; Kennedy et al., 2010; Maes and Twisk, 2010; Tomic et al., 2012
Mitochondrial dysfunction and damage to mitochondria;	Behan et al., 1991; Pietrangelo et al., 2009; Booth et al., 2012; Meeus et al., 2013
Hypovolemia, diminished cardiac output and	Streeten and Bell, 1998; Hurwitz et al., 2009; Miwa and Fujita, 2009; Hollingsworth et al., 2012
blood and oxygen supply deficits to muscles and brain,	McCully and Natelson, 1999; Biswal et al., 2011; Ocon, 2013
especially in an upright position and during exercise;	LaManca et al., 1999; Peckerman et al., 2003; Wyller et al., 2007; Patrick Neary et al., 2008
Reduced (maximum) oxygen uptake;	Farquhar et al., 2002; Weinstein et al., 2009; Vermeulen et al., 2010; Jones et al., 2012
Neurological abnormalities;	Lange et al., 2005; Chen et al., 2008; Puri et al., 2012; Natelson, 2013
Hypocortisolism/blunted hypothalamic-pituitary-adrenal (HPA) axis response;	Demitrack et al., 1991; Lorusso et al., 2009; Papadopoulos and Cleare, 2011; Tak et al., 2011
Ion channel dysfunction (channelopathy);	Watson et al., 1997; Whistler et al., 2005; Broderick et al., 2006; Cameron et al., 2007
A deviant physiological responses to exertion	Thambirajah et al., 2008; Jones et al., 2012; Light et al., 2012; Smylie et al., 2013; Snell et al., 2013

Various of the abnormalities mentioned in Table 2 are reflected by deviant gene expression (Kaushik et al., 2005; Whistler et al., 2005; Fuite et al., 2008; Gow et al., 2009). Aberrations at the cellular level include mitochondrial dysfunction (Booth et al., 2012), inflammation, immunosuppression, e.g., reduced Natural Killer cell lytic activity (Hardcastle et al., 2014), immune dysfunction, e.g., increased synthesis of (Th2-associated) cytokines (Fletcher et al., 2009), increased production of inducible nitric oxide (NO) synthase by peripheral lymphocytes (Maes et al., 2007b). Tissue anomalies encompass significantly low oxygen uptake by muscle cells (Vermeulen and Vermeulen van Eck, 2014), increased intramuscular acidosis after maximal voluntary contraction with significantly prolonged pH recovery times (Jones et al., 2012), an inverse correlation between increased skeletal muscle pH and reduced cerebral blood flow at rest and during dynamic stimulation (He et al., 2013), and differential expression of genes with key roles in mitochondrial function and oxidative balance in the vastus lateralis muscle (Pietrangelo et al., 2009). Neurological abnormalities in ME/CFS patients or subgroups thereof include reduced gray and white brain matter (Puri et al., 2012), increased magnetic resonance imaging (MRI) abnormalities, mainly T2 signal hyperintensities in the frontal lobes (Lange et al., 1999), significant reductions in cerebral blood flow across various brain regions (Yoshiuchi et al., 2006), spinal fluid abnormalities (Natelson et al., 2005) and increased ventricular lactate (Shungu et al., 2012).

Since many of the research studies which observed aberrations in ME/CFS compared patients with sedentary controls, the abnormalities cannot be attributed to deconditioning. Whether the name ME is appropriate or not remains subject of debate (Van der Meer and Lloyd, 2012), but various findings in ME/CFS actually indicate muscular and neurological abnormalities. However, immunological abnormalities, antigens, increased oxidative and nitrosative stress, and gastro-intestinal dysfunction seem to be at the core of the etiology of ME/CFS (Maes and Twisk, 2010). The distinction between patients meeting the proposed criteria for ME (Carruthers et al., 2011) and patients with CFS (Fukuda et al., 1994) not fulfilling these criteria is reflected by specific immunological abnormalities (Brenu et al., 2013).

Exercise and orthostatic stress seems to induce or intensify (long lasting) abnormalities in patient subgroups, e.g., a significant decrease of the oxygen consumption and workload at exhaustion and at the ventilatory (anaerobic) threshold at a second exercise test 24 h later (Snell et al., 2013); a long-term increase of messenger RNA (mRNA) of metabolite-detecting receptors after sustained moderate exercise (White et al., 2012); a significant prolongation of the time taken for pH to recover to baseline after exercise (Jones et al., 2012); reduced prefrontal oxygenation during exercise and recovery when compared to healthy controls similar in physical activity levels (Patrick Neary et al., 2008); impaired cognitive functioning 24 h after physically demanding exercise compared with sedentary healthy individuals (LaManca et al., 1998); and an substantially higher increase in NO metabolites in relation to workload during exercise (Suárez et al., 2010).

Although the exact etiological mechanisms remains to be elucidated, the post-exertional malaise phenomenon could arguably be explained by the observation that physical stress, particularly anaerobic exercise, can intensify pre-existing abnormalities: inflammation (Fisher-Wellman and Bloomer, 2009; Mogharnas et al., 2011; Sakharov et al., 2012), immunosuppression (Millard et al., 2013), immune dysfunction (Steensberg et al., 2001), oxidative and nitrosative stress (Bloomer et al., 2005; Fisher-Wellman and Bloomer, 2009; Sakharov et al., 2012) and hyperpermeability of the intestines (Lambert, 2009; Lamprecht and Frauwallner, 2012). Since the anaerobic threshold is already low in ME/CFS patients or subgroups (De Becker et al., 2000; Vermeulen et al., 2010), the effects of exercise programmes should be monitored objectively and continuously, e.g., by using CPET.

## Subgroups

### Clinical subgroups

In addition to a breakdown into patients with post-exertional malaise (ME) and without this hallmark feature (CFS/not ME), a subdivision into subgroups based upon onset (sudden/viral? or gradual), type and severity of symptoms, gender, age, duration and/or comorbidities seems inevitable to unravel common and distinct deviant biological pathways in more detail. Potentially relevant clinical subdivisions are summarized in Table S1 (see Supplementary Material).

### Biological subgroups

Several studies have revealed distinct biological ME/CFS patient subgroups, often associated with distinct differences in the clinical picture as illustrated in Table S2 (Supplementary Material). The most relevant subdivisions are based upon (exercise-induced) immunological abnormalities. Acknowledging immunological, infectious and endocrine subdivisions seem to be crucial to establish the efficacy and safety of pharmacological and behavioral therapies. In addition to differential gene expression genes in all patients, upregulation and downregulation of specific genes are likely to reflect specific (biological and clinical) ME/CFS patient subgroups.

## Discussion

Although ME and CFS are considered to be equivalents (WHO, 1992), the diagnostic criteria for ME (Ramsay, 1988; Dowsett et al., 1990; Carruthers et al., 2011; Jason et al., 2012a) and CFS (Sharpe et al., 1991; Fukuda et al., 1994; Reeves et al., 2005) define distinct clinical, partly overlapping, entities. CFS criteria focus primarily on chronic fatigue, which, due to is nature, is a subjective and ambiguous criterion (Jason et al., 2010, 2011a; Davenport et al., 2011). ME is principally characterized by neurocognitive impairment, (muscle) weakness and sleep disturbances, but the distinctive feature of ME is post-exertional “malaise”: a (long-lasting) aggravation of symptoms, e.g., pain, “brain fog” and weakness, after a minor physical or mental exertion (Carruthers et al., 2011; Maes et al., 2012a).

The question whether the label ME is appropriate (Baraniuk et al., 2005; Schutzer et al., 2011) or not remains to be established, but considering the confusion due to the introduction of chronic fatigue as the principle criterion and the use of symptom-based criteria and questionnaires in clinical practice and research, it seems crucial to assess various characteristic symptoms objectively and to make a clear distinction between patients with post-exertional malaise (ME) and patients without post-exertional malaise. Objective tests, e.g., repeated exercise tests (CPETs) and tilt table testing, could be employed as a solid basis for the diagnosis, the validation, adjustment and refinement of ME (Carruthers et al., 2011) and CFS (Fukuda et al., 1994) criteria and the definition of clinical ME and CFS patients subgroups in research.

Trials into the efficacy and safety of pharmacological (Fluge et al., 2011; Watt et al., 2012) and behavioral therapies, e.g., CBT and GET (Knoop et al., 2007; Heins et al., 2010; White et al., 2011), should employ objective measures of the clinical status and improvement thereof and biomarkers to establish the effects of these therapies in specific clinical and biological subgroups (Kindlon, 2012). To unravel the enigma and to resolve the controversy, patients should be monitored before, during and after potentially effective therapies in research and clinical practice using objective measures (Kindlon, 2012), e.g., oxygen uptake at the anaerobic threshold and maximum oxygen uptake (VO2max), and biomarkers, e.g., (exercise-induced) cytokine levels.

## Conclusion

Looking at the definitional criteria of ME, e.g., (muscle) weakness, cognitive impairment, but above all post-exertional “malaise,” ME is not equivalent to CFS, let alone chronic fatigue. The introduction of the label CFS, but more importantly a fatigue-based case definition, have resulted into research of a heterogeneous patient population of patients with chronic fatigue. Combined with the use of subjective measures this has created confusion and controversy.

Several authors have questioned the validity and nature of symptoms reported by patients. This debate can be resolved by assessing characteristic symptoms using objective methods, if possible, e.g., repeated exercise tests, cognitive tests, orthostatic testing and visual tests.

Despite the ambiguous CFS criteria and methods applied, researchers have observed various specific abnormalities repeatedly, e.g., (reactivating or chronic) infections, immunological anomalies (inflammation, immune activation and dysfunction), and long lasting deviant effects of exercise, in the ME/CFS patient population or substantial subgroups thereof.

However, in order to advance in research and clinical practice, to unravel the enigmatic cause(s) of ME and CFS and to develop effective therapies, it seems crucial (a) that patients with post-exertional “malaise” (ME) and “CFS” patients without post-exertional phenomena are acknowledged as two separate clinical and research entities; (b) that typical symptoms of ME and CFS are assessed objectively as much as possible; (c) that well-defined clinical subgroups of ME and CFS, e.g., patients with orthostatic intolerance or patients with sudden-onset, are investigated in more detail; (d) that biomarkers, e.g., immunological status in rest and after exertion, are used to distinguish biological subtypes in research; and (e) that trials into the efficacy of therapies use objective measures of the clinical status and biomarkers to establish the effects of these therapies in ME or CFS patients or subgroups thereof impartially, e.g., by a (positive) change in the oxygen uptake at the anaerobic threshold and cognitive tests scores.

Considering the central role of post-exertional and cognitive impairment (“brain fog”) in ME/CFS, interesting areas of research include exercise-induced abnormalities, e.g., immune responses, oxidative and nitrosative stress, oxygen uptake, mitochondrial dysfunction, and brain energetics before, during and after exercise and cognitive stimulation (Quistorff et al., 2008; Bergersen and Gjedde, 2012; Shungu et al., 2012).

### Conflict of interest statement

The author declares that the research was conducted in the absence of any commercial or financial relationships that could be construed as a potential conflict of interest.

## Footnotes

^1^ME/CFS refers to ME and/or CFS.
