# Supplementary material for: The status of and future research into Myalgic Encephalomyelitis and Chronic Fatigue Syndrome: the need of accurate diagnosis, objective assessment, and acknowledging biological and clinical subgroups
Source: Front Physiol. 2014 Mar 27;5:109. doi: 10.3389/fphys.2014.00109 (PMC3974331; doi:10.3389/fphys.2014.00109)
Supplement: Supplementary file 1 [file Presentation1.PDF]

Appendix

Table 3.
Potentially relevant clinical subgroups.

Criterion	Observation(s)	Reference
Illness onset: gradual (viral) or sudden	Immunological abnormalities are indicative for a viral (infectious) nature of sudden-onset ME/CFS.	Komaroff, 1988; Komaroff, 1994; Reyes et al., 1998
	Distinct patterns of immune activation in sudden-onset/viral ME/CFS patients and non-viral patients.	Porter et al., 2010
	Increased interleukin (IL)-8 levels in the spinal fluid of patients with sudden (viral-like) onset.	Natelson et al., 2005
	A higher prevalence of comorbid psychiatric disease in the gradual onset patient group.	DeLuca et al., 1997
	More severe cognitive deficits in the sudden-onset patients group.	DeLuca et al., 1997; Claypoole et al., 2007
Duration of the illness and age	HPA axis abnormalities seem to be more prevalent at latter stages of the disease.	Cleare 2004; Gaab et al,. 2004
	Patients with long-duration illness report more and more severe specific cognitive difficulties than patients with short-duration ME/CFS.	Friedberg et al., 2000
	A negative association between recovery rates and the duration of the illness.	Clark et al., 1995; Ray et al., 1997; van der Werf et al., 2002; Jason et al., 2011; Reyes et al., 1998
Presence and severity of specific symptoms	Significantly lower stroke volume and cardiac output in patients with “severe” ME/CFS compared to less-severe ME/CFS and controls.	Peckerman et al., 2003
	Postexertional malaise and flu-like symptoms differentiate patients with “severe” ME/CFS and are highly predictive of lower cardiac output.	Peckerman et al.; 2003
	Significantly higher prevalences of a lower left ventricular end-diastolic mass and cardiac index in ME/CFS patients with orthostatic intolerance.	Miwa and Fujita, 2011
	An association of clinical phenotypes with clusters of differentially expressed genes.	Kerr et al., 2008
Comorbid conditions	Abnormal neuropsychological test results in ME/CFS patients without fibromyalgia not observed in ME/CFS patients with comorbid fibromyalgia.	Cook et al., 2005
	Higher increases in specific metabolite-detecting receptors in response to sustained moderate exercise in patients with comorbid fibromyalgia, in addition to mRNA increases in other metabolite-detecting receptors and adrenergic receptors in all ME/CFS patients.	White et al., 2012
	Patients without a psychiatric diagnosis score worse on tests of memory, attention, and information processing than patients with a psychiatric disorder.	DeLuca et al., 1997
	More areas of reduced blood flow in patients without a comorbid psychiatric condition compared to the patients with a concurrent psychiatric diagnoses.	Yoshiuchi et al., 2006
	A larger number of brain abnormalities on T2 weighted MRI images in patients without a psychiatric diagnosis compared to the patient group with a psychiatric diagnosis.	Lange et al., 1999
Pre-illness history and triggers	An association between pre-illness sport levels and/or episodes of severe acute infection and oxidative stress and suppression of protective heat shock proteins in response to exercise.	Jammes et al., 2012
	ME/CFS can be instigated by various (immunological) triggers.	De Becker et al., 2002

Table 4.
Potentially relevant biological subgroups.

Subgroups	Observation(s)	References
Immunological	Patients with low Natural Killer cell activity (NKCA) report more daytime dysfunction, and exhibit more cognitive deficits, than patients with normal NKCA.	Siegel et al., 2006
	Patients can be subdivided in two mainly non-overlapping subgroups with specific immunological abnormalities, in addition to common immunological aberrations.	Patarca et al., 1995
	Patients with more extensive immune activation and dysfunction report more severe cognitive deficits.	Lutgendorf et al., 1995
	Th2-predominant immune activation is observed in a (large) subgroup only.	Fletcher et al., 2009; Porter et al., 2010; Roelant and de Meirleir, 2012
	Differential gene expression in post-viral ME/CFS patients indicate abnormalities in immune modulation, oxidative stress and apoptosis.	Gow et al., 2009
Infectious	The presence of specific infections and antigens seem to predict the outcome of pharmacological interventions.	Montoya et al., 2013; Lerner et al., 2010
Genomic	Differentially expressed genes in seven patient groups are associated with distinct differences in symptomology, severity and limitations.	Kerr et al., 2008
	Subtype-specific relationships of differential gene expression with elevated antibodies to Epstein-Barr virus (EBV) and enteroviruses.	Zhang et al., 2010
Endocrine	Specific HPA axis abnormalities, hypocortisolism and blunted cortisol response to adrenocorticotropic hormone (ACTH) etc., are observed in patient subgroups only and seem to be associated with the duration of the illness.	Gaab et al., 2004; Scott et al., 1998; Parker et al., 2001
	A reduction of the adrenal gland bodies by over 50%, indicating significant adrenal atrophy, in patients with a subnormal cortisol response to ACTH.	Scott et al., 1999
	A significant association of the nocturnal adrenal response to ACTH and cortisol feedback to the HPA axis with specific somatic symptoms.	Aschbacher et al., 2012
Neurological	Patients with MRI identified brain abnormalities report being more physically impaired.	Cook et al., 2001
	Cerebral changes in patients without psychiatric comorbidities consist mostly of small, punctate, subcortical white matter hyperintensities, predominantly in the frontal lobes.	Lange et al., 1999
	A significant association between ventricular lactate and physical health and disability indices.	Shungu et al., 2012; Mathew et al., 2009
Exercise-response	Gene expression after moderate exercise implicate two subgroups: patients with long-lasting increased gene transcription for various sensory and adrenergic receptors, and patients with decreased transcription of α-2A adrenergic receptors.	Light et al., 2012
	The severity of post-exertional malaise is related to an increase in cytokine levels after moderate exercise.	White et al., 2010
	Low-level bouts of plantar flexion contractions indicate two groups: patients with normal phosphocreatine (PCr) depletion, increased intramuscular acidosis and prolongation of pH recovery times, in response to exercise, and patients with low PCr depletion.	Jones et al., 2012
	In addition to exercise-induced immunological gene expression abnormalities in the ME/CFS patient group, exercise seems to provoke sex-specific immune responses in ME/CFS.	Smylie et al., 2013
Metabolic	Based upon the pathways employed to compensate for mitochondrial dysfunction two major subgroups can be differentiated.	Booth et al., 2012; Myhill et al., 2009
